# Effect of Adhesive Debonding on the Performance of Piezoelectric Sensors in Structural Health Monitoring Systems

**DOI:** 10.3390/s19235070

**Published:** 2019-11-20

**Authors:** Xuerong Liu, Yuanming Xu, Ning Li, Xiangyu Wang, Weifang Zhang

**Affiliations:** 1 School of Aeronautic Science and Engineering, Beihang University, Beijing 100191, China; 2 School of Energy and Power Engineering, Beihang University, Beijing 100191, China; 3 School of Reliability and Systems Engineering, Beihang University, Beijing 100191, China

**Keywords:** debonding area, PZT discs, adhesive, structural health monitoring

## Abstract

Piezoelectric (PZT) ceramic elements are often subjected to complex loads during in- service lifetime in structural health monitoring (SHM) systems, and debonding of both excitation actuators and receiving sensors have a negative effect on the monitoring signals. A first systematic investigation of debonding behaviors by considering actuators and sensors simultaneously was performed in this paper. The debonding areas of actuators were set in different percentage range from 0% to 70%, and sensors in 0%, 20%, 40% and 60%. The signal-based monitoring method was used to extract the characteristic parameters of both the amplitudes and phases of received signals. Experimental results revealed that as the debonding areas of the actuators increase, the normalized amplitude appears a quick decrease before 35% debonding area of actuators and then a slow rise until 60% of debonding reached. This may be explained that the 35% debonding turning point correspond to the coincidence of the excitation frequencies of peripheral actuators with the inherent frequency of the central piezoelectric sensor, and the 60% be the result of the maximum ability of piezoelectric sensor. The degrees of debonding of actuators and sensors also have significant influence on the phase angle offset, with large debonding of actuators increases the phase offset sharply. The research work may provide useful information for practical monitoring of SHM systems.

## 1. Introduction

As a real-time monitoring technology for detecting structural damage and aging, structural health monitoring (SHM) technology is widely used in aerospace, civil engineering, machinery, transportation, and other fields [1,2]. The piezoelectric (PZT) actuator/sensor network is considered as the most reliable and visible option for SHM system, and piezoelectric elements are usually fixed on the structure by the interface adhesive layer, which plays the role of forces and strains transferring between piezoelectric components and structures [3,4]. Therefore, the integration of driving and sensing functional components with host structures is the premise of structural health monitoring technique. In the actual service environment, the piezoelectric components often subjected to similar environmental conditions as those of the structure, including the combined effects of temperature change, mechanical load, humidity, salt spray, etc. [5,6,7]. It often causes debonding of piezoelectric elements as a result of the environmental factors and the existing adhesive technology. Therefore, investigating the adhesive debonding behavior of piezoelectric actuator/sensor is particularly important to improve the accuracy of damage assessment in structural health monitoring systems.

Many structural damage detection researches have been carried out using PZT elements [8,9,10,11,12,13], but insufficient attention has been paid to the damage or failure of PZT element, especially partial debonding damage for both the exciting actuators and receiving sensors, (i.e. most researches only studied either the debonding of actuators or sensors). There are two main methods to detect the damage in structures, one iselectro-mechanical impedance technique, the other is wave propagation method. [14,15,16,17]. Park et al. [18] described the influence of bonding defects between PZT discs and matrix structures through Lamb wave propagations and impedance methods. It has shown that debonding can significantly affect the amplitude and phase, as well as alter the impedance spectrum. And a debonding identification algorithm was proposed to diagnose the degradation of the mechanical and electrical performance of PZTs and the debonding flaws between PZTs and structures. Qing et al. [19] researched the influence of the adhesive thickness and its modulus on the behaviors of piezoelectric discs by electromechanical impedance method. The results showed that the thickness of adhesive would change the electromechanical impedance and the amplitude of the sensor signal. The mechanical impedance and signal response to modulus of piezoelectric sensor were different at high and low frequencies. The elastic wave propagation process was studied bythrough experimental analysis and computational model methods when the sensor was on debonding state [20]. The results showed a significant performance loss when the debonding percentage of sensor increased. Lanzara [21] et al. conducted experimental and numerical study to analyze the effect of interface debonding behavior on the performance of PZT sensors. The amplitude and signal delay were studied by changing the debonding area, bonding shape, and location underneath the PZTs. 2D spectral element simulation method was used to verify the experimental results. 

Although many previous works have that the debonding area, adhesive thickness and modulus of adhesive have influence on the monitoring system. However, few attentions have been focused on both the debonding of exciting actuators and receiving sensors, while both the debonding of actuators and sensors may occur during service. Nevertheless, thereis no systematic study on the debonding area of actuators and sensors, especially from small-area debonding up to large-area debonding, and the mechanism of monitoring signal increasing with the debonding area of piezoelectric elements is unclear. Hence, the debonding behavior of both actuators and sensors requires comprehensive investigation. 

In this paper, a systematic experimental investigation of both the debonding of actuators and sensors was designed. The debonding areas of actuators were set from 10% to 70%, the debonding areas of receiving sensors were 0%, 20%, 40% and 60%, the excitation frequencies were 50 kHz, 60 kHz and 70 kHz. Aluminum 2024-T3 was chosen as base plate. Signal-based damage monitoring method was used to extract the characteristic parameters of normalized amplitude and phase difference ofA0 mode Lamb wave on the basis of these results. And the influence of both the debonding behavior of actuators and sensors onmonitoring signal was analyzed. 

## 2. Theory of Piezoelectric Element Debonding Based on Lamb Wave Propagation

### 2.1. Lamb Wave Propagation in Aluminum Plate

**Figure 1:**
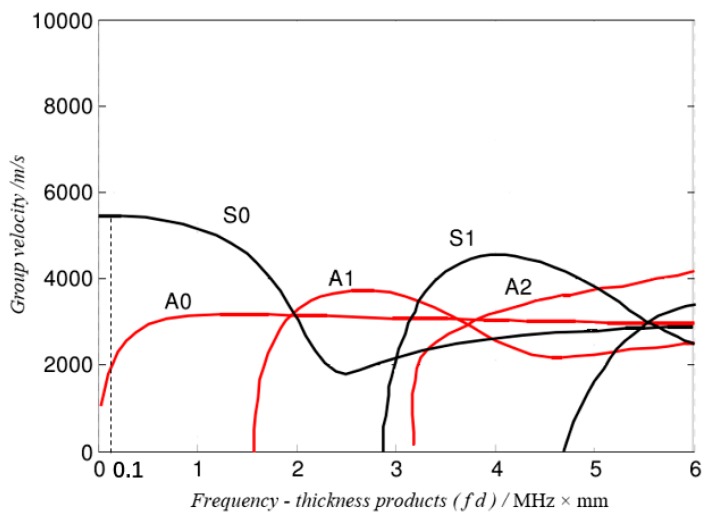
Dispersion curve of Lamb wave propagating in aluminium alloy plate.

Lamb waves was first used for structural health monitoring technology by the US general engineer Worlton [22]. Transverse wave and longitudinal wave are two types of waves that exist only in an infinitely uniform, isotropic elastic medium. When the waves propagate in the aluminum alloy plate, a wave containing a large number of wave packets is formed, such waves are called Lamb waves [23]. Lamb waves are dispersive waves. Figure 1 is the group velocity dispersion curve of Lamb wave propagating in a 2 mm thick aluminium alloy plate. Lamb waves can be classified into two modes: symmetric (S) mode and anti-symmetric (A) mode. Each mode contains multi-order patterns, symmetric mode includes S0, S1, …, Sn, etc., anti-symmetric mode includes A0, A1, …, An, etc. In order to reduce the influence of the dispersion characteristics of the Lamb wave, the frequency-thickness product (*f·s*) was chosen to be 0.1 MHz*mm in Figure 1, so only A0 mode and S0 mode waves propagated in the aluminium alloy plate. The propagation speed of the S0 mode wave is much larger than the A0 mode wave, therefore, the first wave packet is the S0, and the second wave packet is the A0. The A0 mode wave was applied for piezoelectric element debonding given its higher energy and signal to noise ratio. 

**Figure 2:**
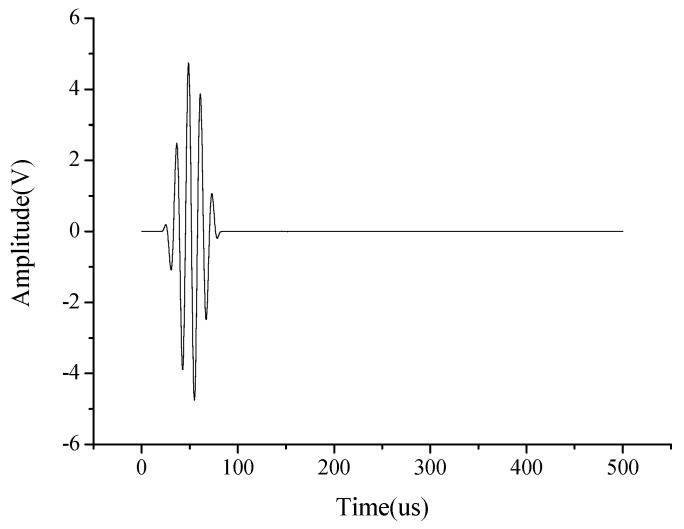
Excitation signal of 5 cycles and 50 kHz actuation frequency.

The number of cycles for each excitation pulse of the excitation signal is generally 3.5–13.5 cycles [24]. The number of cycles should not be too large or too small, the more period will lead to crosstalk in different mode of wave packet, while small signal cycles carrid less energy. Besides, the wider the bandwidth, the signal will be susceptible to interference. In this study, five cycles of the sinusoidal narrowband signal modulated by Hanning window (as shown in Figure 1) was selected as the excitation pulse [25,26], because the sinusoidal signal has periodicity, smoothness and peak time is faster than the parabolic shape, and the narrowband signal is easier to interpret than the broadband signal insignal analysis. The excitation frequency is set to be 50 kHz, as shown in Figure 2. The input signal is 5 V and the maximum output voltage is 50 V, the high-speed elastic wave excitation module containing power amplifier fixed 10 times to amplify the input signal.

(1) $$u\left(t\right)\;=\;A[H\left(t\right)-H\left(t-\frac{N}{f_{c}}\right]\times \left(1-cos\frac{2\pi f_{c}t}{N}\right)sin2\pi f_{c}t$$

where A is the amplitude of the signal, $f_{c}$ is the center excitation frequency, N is the number of excitation signal cycles, and H(t) is the Heaviside step function.

**Figure 3:**
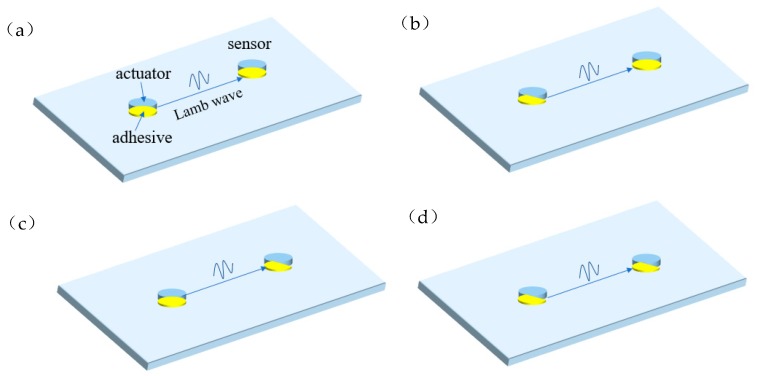
Propagation of Lamb wave on aluminum plate using pitch catch mode: (**a**)excitation actuator and the receiving sensor are not debonded; (**b**) only actuator is debonded; (**c**) only receiving sensor is debonded; (**d**) both actuator and sensor are debonded.

SHM technology can be divided into active SHM and passive SHM; the active SHM is widely used to directly asses the structure health status. Piezoelectric elements are used to build a structural health monitoring network. There are two modes of damage monitoring using piezoelectric elements, one is pulse-echo mode and the other is active pitch-catch mode [27,28]. In this study, pitch-catch mode is used in debonding damage monitoring. The active Lamb wave signals were generated by driving actuators, then propagated in the structure and received by the sensors. Figure 3 is a schematic diagram of the propagation of Lamb wave on the aluminum plate. There are four cases of piezoelectric element debonding. Figure 3a shows that the excitation actuator and the receiving sensor are not debonded, Figure 3b represents that only actuator is debonded, Figure 3c shows that only receiving sensor is debonded, Figure 3d shows that both the actuator and sensor are debonded.

Due to the positive piezoelectric effect and inverse piezoelectric effect, the piezoelectric materials can be made into piezoelectric sensors and actuators, which can be used to monitor the charge density on piezoelectric dielectrics and to change the structural deformation or stress state, the charge density on piezoelectric dielectrics is proportional to the external force. The constitutive equation of piezoelectric materials is as follows.

(2) $$\varepsilon_{ij}\;=\;s_{ijkl}^{E}\sigma_{kl}+d_{kij}^{c}E_{k}$$

(3) $$D_{j}\;=\;d_{jkl}^{d}\sigma_{kl}+e_{jk}^{\sigma }E_{k}$$

where $\varepsilon_{ij}$ is the mechanical strain, $D_{j}$ is the electric displacement, $E_{k}$ is the electric field and $\sigma_{kl}$ is the mechanical stress, $e_{jk}^{\sigma }$ represent the dielectric constant under constant stress, $s_{ijkl}^{E}$ is the coefficient of flexibility under constant electric field, $d_{kij}^{c}$ and $d_{jkl}^{d}$ is piezoelectric voltage constant.

On the receiving sensor, due to the debonding of piezoelectric components, the contact area between the piezoelectric sensor and the substrate decreases, irrespective of other factors that cause the charge density of piezoelectric sensors to change, which will result in thedecrease of the accumulated charges on the receiving sensor, leading to the decrease of the energy of the received signal and the signal amplitude. 

### 2.2. Monitoring System Setup for Debonding Tests

**Figure 4:**
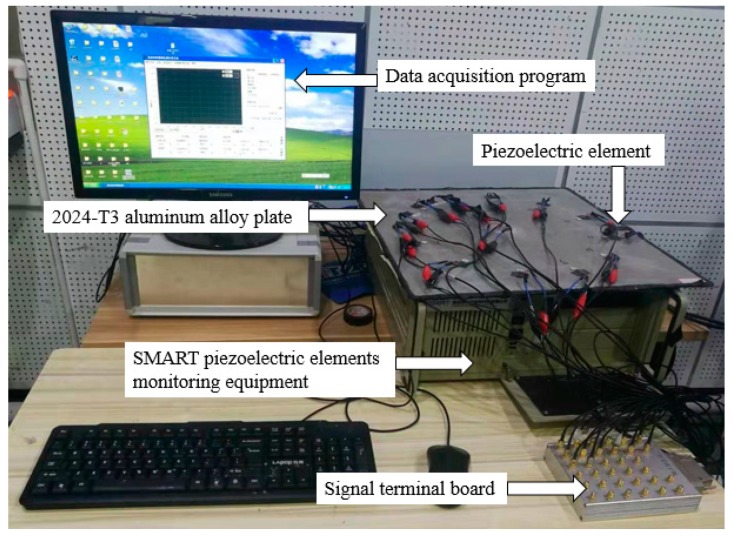
Establishment of structural health monitoring system for piezoelectric elements.

The Integrated Structural Health Monitoring Scanning System (SHM-ISS-4.0A), which was provided by Nanjing SMART Monitoring Technology Co., Ltd. was used to excite and receive signals when the PZT elements are in different percentages of debonding. As shown in Figure 4, the entire PZT debonding monitoring system is composed of SHM-ISS-4.0A system (including data acquisition program and SMART piezoelectric element monitoring equipment, the equipment is composed of high speed elastic wave excitation and response module, high speed elastic wave excitation response channel scanning module, as well as various interfaces and heat dissipation devices), signal terminal board and 2024-T3 aluminum alloy plate with different degrees of debonding piezoelectric pieces. The system integrates various functions such as structural state analysis, damage characteristic parameters, it is a highly integrated structural health monitoring system that is suitable for both industrial field applications and scientific research. So in this study, the system can be used to extract the characteristic parameters of amplitude and phase shift of monitoring signals.

**Figure 5:**
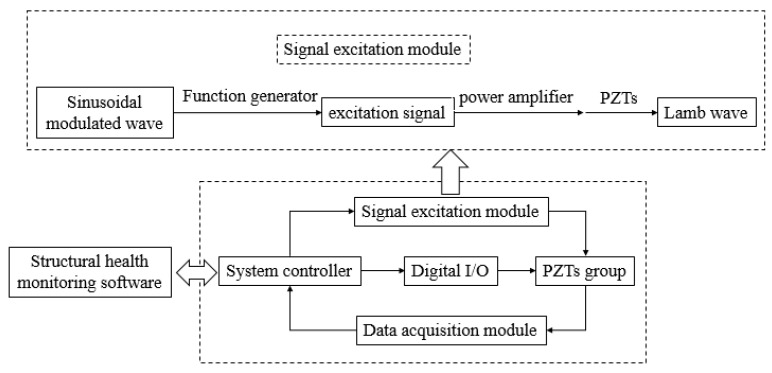
Schematic diagram of the piezoelectric sensor debonding monitoring system. I/O = Input/Output.

The debonding monitoring system of the piezoelectric sensor utilizes the active monitoring method of pitch catch mode, and the response variable takes the amplitude and phase difference of the signal received by the sensors. The schematic diagram of the piezoelectric element debonding monitoring system is shown in Figure 5. In the signal excitation module, the sinusoidal modulation wave generates a specific excitation signal through a function generator. The excitation signal is amplified by a power amplifier, and thenLamb wave is generated by the inverse piezoelectric effect of the piezoelectric actuator. Lamb wave propagates to the sensor through the structure. The sensor receives stress wave through piezoelectric effect. Then the data are collected through the data acquisition module. In this system, parameters can be set by system controller and monitored by structural health monitoring software.

### 2.3. Extraction of Characteristic Parameters

**Figure 6:**
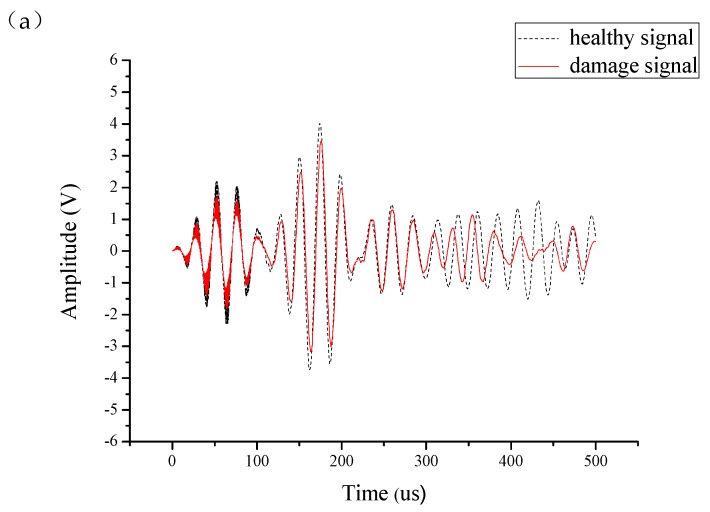

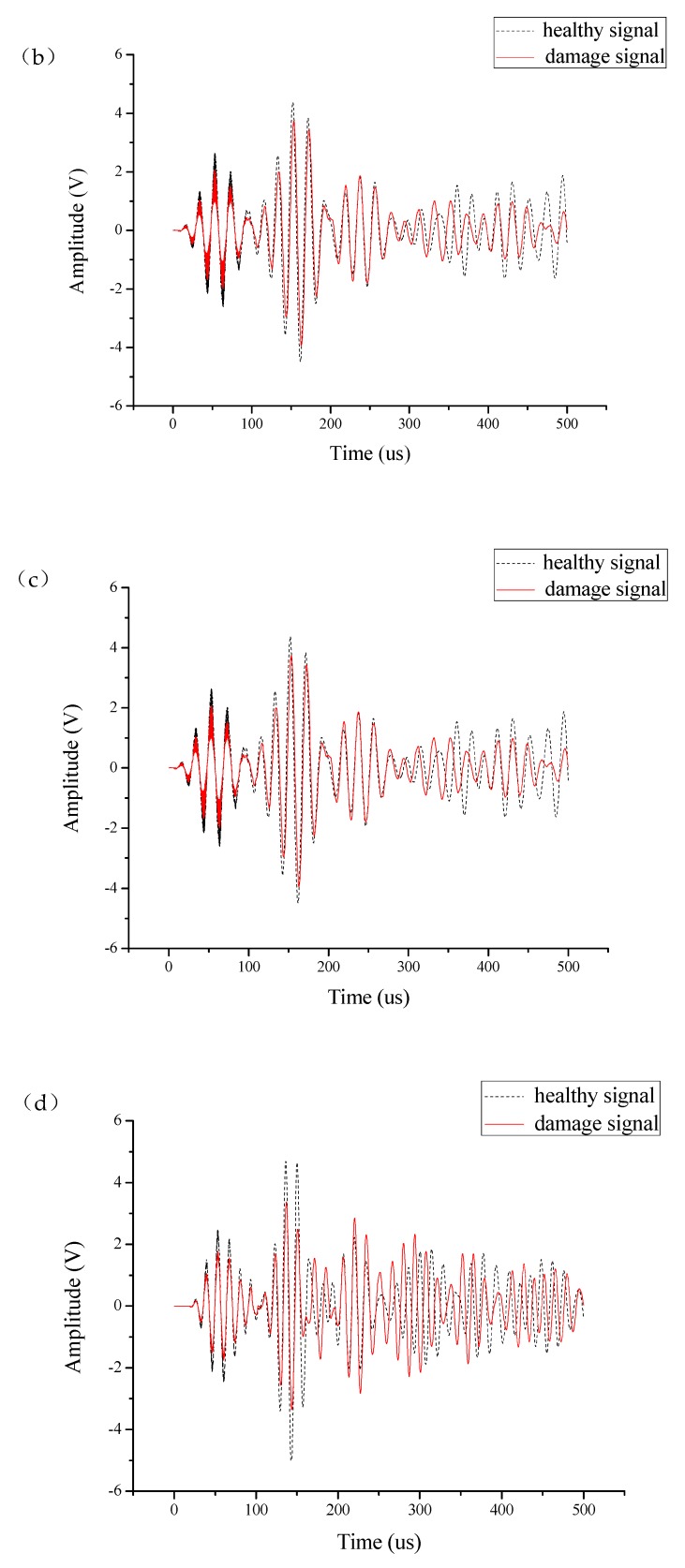
Piezoelectric sensor monitoring results with different excitation frequencies: (**a**) 40 kHz; (**b**) 50 kHz; (**c**) 60 kHz; (**d**) 70 kHz.

As shown in Figure 6, the dotted line represents the healthy signal when the piezoelectric discs are full bonded on the aluminum plate, the solid line represents the damage signal when the debonding area of actuator is 20%. The comparison of the sensing signals obtained by different excitation frequencies for partial debonding and full bonded piezoelectric elements shows that when the frequency thickness product (f·d) is 0.1 MHz*mm (the thickness of aluminum plate is 2 mm), the A0 mode signal is more sensitive to the change of debonding area. Therefore, 50 kHz and A_0_ mode are chosen as the excitation frequency and signal mode to monitor the signal changes of piezoelectric sensors under different debonding area conditions.

**Figure 7:**
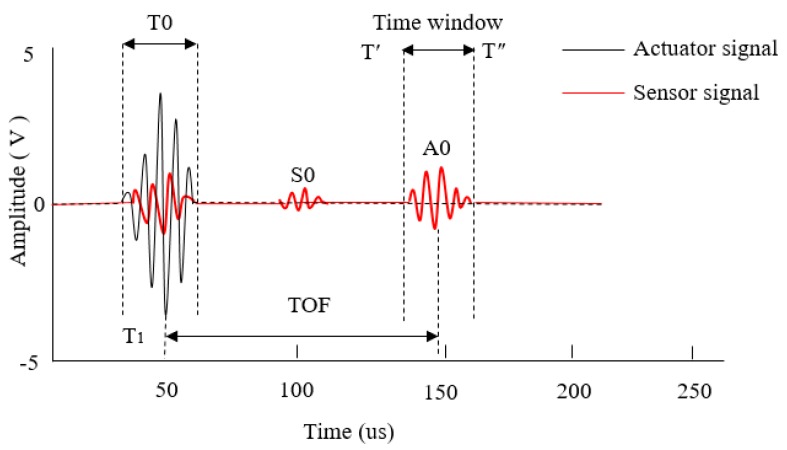
Intercepting the A0 mode wave packet of Lamb wave using an actuator signal (in black) and a sensor signal (in red).

Figure 7 is a schematic diagram of the time window of the A0 mode wave packet intercepting the Lamb wave, the black line represents the excitation signal and the red line represents the received signal. T_0_ is the duration of the excitation signal propagation, and TOF is the flight time of the signal from an actuator to a sensor, the measurement standard for the extraction of TOF is based on the arrival time of the maximum peak of A0 mode Lamb wave. 

In order to investigate the degradation trends of PZT actuator/sensor under different percentages of debonding, the method of extracting characteristic parameters of the signal was performed. [29,30] There are two main characteristic parameters ofLamb wave signal during the debonding process of actuator/sensor, one is normalized amplitude, which corresponding to the energy of the Lamb wave, the other is phase angle offset ofreceiving signal, which represents the propagation path of Lamb wave [31]. During the debonding process of the piezoelectric sensor, the energy and propagation path will change, which leads to the changes of normalized amplitude and phase angle offset respectively.

To simplify the calculation, the absolute value of the physical system was changed into relative value. The amplitude of the acquired Lamb wave signal is normalized as following formula:

(4) $$x=\left|\frac{A_{i}}{A_{0}}\right|$$

In Equation (4), x represents the normalized amplitude, $A_{i}$ represents the amplitude of the wave packet signal in the ith case, and $A_{0}$ represents the amplitude of the wave packet in the reference signal (the initial state of the piezoelectric element). 

The phenomenon of partial debonding or even shedding of the PZT discs may occur in actual environment, such as vibration, which may lead to the change of propagation distance of Lamb wave, i.e. the phase angle offset. The formula for calculating the relative phase angle shift is as follows:

(5) $$y=\frac{p_{i}-p_{0}}{p_{0}}$$

In the Equation (5), $P_{i}$ represents the time corresponding to the maximum amplitude of $A_{0}$ wave packet in the i-th case, and $P_{0}$ denotes the time corresponding to the maximum amplitude of the $A_{0}$ wave packet of the reference signal (in the initial state of the piezoelectric element).

## 3. Debonding Test Design

### 3.1. Debonding Design

**Figure 8:**
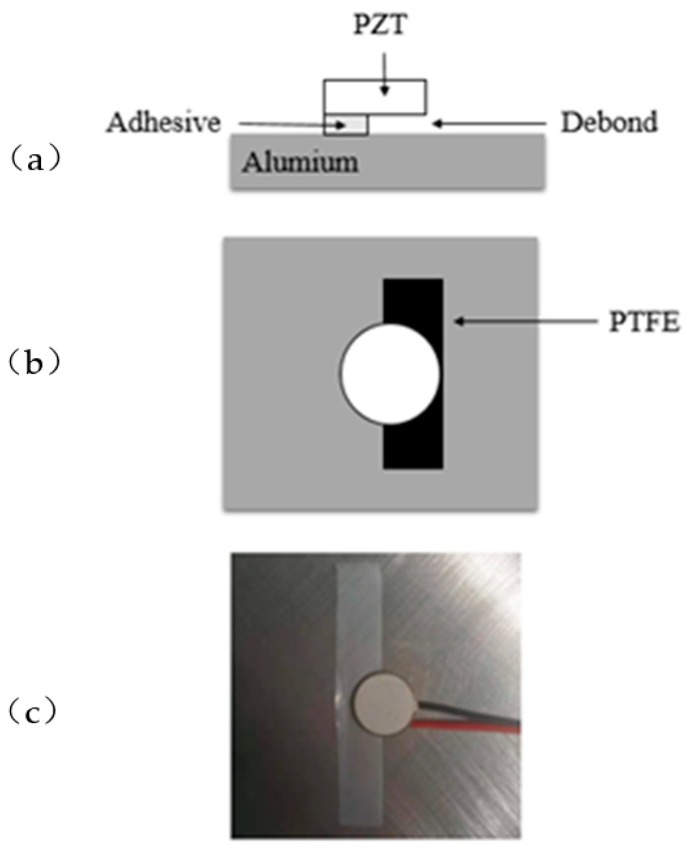
Control method of debonding area and adhesive thickness: (**a**) positive view image; (**b**) top view image; (**c**) actual diagram.

**Figure 9:**
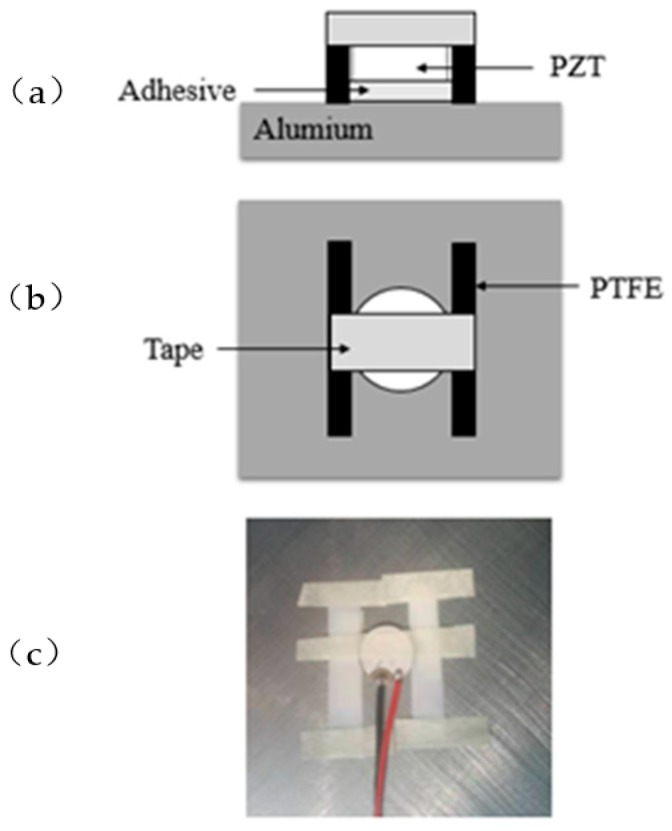
Control method of adhesive thickness without debonding: (**a**) positive view image; (**b**) top view image; (**c**) actual diagram.

The debonding area of piezoelectric elements and the thickness control of adhesive are two important factors study the debonding effect of piezoelectric sensor. Figure 8 and Figure 9 are the schematic diagrams of the control methods of debonding area and adhesive thickness when the PZT discs at partial debonding and full bonded state, respectively. As shown in Figure 8, Figure 8a is the positive view image, Figure 8b is the top view image, Figure 8c is actual diagram. The bonded area of the adhesive is the area of piezoelectric disc minus the debonding area caused by PTFE (polytetrafluoroethylene). The thickness of the adhesive is equal to the thickness of PTFE film (which is 0.04 mm). As shown in Figure 9, Figure 9a is the positive view image, Figure 9b is the top view image, Figure 9c is the actual diagram. Among them, the thickness of adhesive is equal to the thickness of PTFE minus the thickness of piezoelectric disc. Because the thickness of piezoelectric disc and PTFE film are 0.6 mm and 0.64 mm, respectively, so thickness of the adhesive is 0.04 mm.

**Figure 10:**
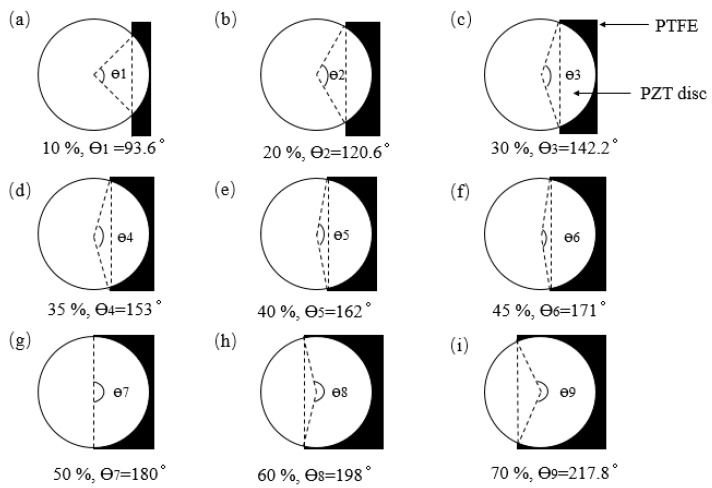
PTFE film controls the debonding area of the piezoelectric sensor. The corresponding debonding areas are: (**a**) 10%; (**b**) 20%; (**c**) 30%; (**d**) 35%; (**e**) 40%; (**f**) 45%; (**g**) 50%; (**h**) 60% and (**i**) 70%.

The polytetrafluoroethylene (PTFE) is a completely non-stick coating, it is widely used as an artificial debonding tool, and because of its lower mechanical properties, the impact of PTFE on the monitoring results is negligible [21,32]. Since the PTFE film can cause a simulated debonding effect in the structure, the debonding area and adhesive thickness of piezoelectric discs are controlled by inserting PTFE film between piezoelectric discs and matrix structure. The PTFE was cut into rectangles to control the debonding area. Figure 10 is a schematic diagram showing the debonding area of a piezoelectric element controlled by PTFE film. When the piezoelectric sensor is fully bonded to the structure, the contact area between the piezoelectric sensor and the substrate is 113.1mm^2^, and this is set as a reference. Debonding area is controlled by adjusting the angle between piezoelectric sheet and PTFE film. As shown in Figure 10, the angles Ɵ_i_ (I = 1, 2, …, 9) corresponding to different degree of debonding are set from 10% to 70%, and the corresponding values of debonding areas were presented in Table 1. 

### 3.2. Debonding Manufacturing Process

The Al 2024-T3 rectangular plate was used in the study, the detailed dimension of the plate was 500 mm long, 500 mm wide and 2 mm thick. The performance parameters of Al 2024-T3 are shown in Table 2. Generally, commonly used piezoelectric elements have rectangular and circular shapes. The circular piezoelectric ones are more conducive to the study of debonding effect. Diameters vary in size from a few millimeters to more than twentieth millimeters. The larger diameter sensors are more effective to the study of debonding area, but the matching of the sensor to the structural matrix and the cost should also be considered. Therefore, the circular piezoelectric discs with diameter of 12 mm isselected. The piezoelectric discs are manufactured by Stem Corporation of the United States. The performance of the discs is shown in Table 3. The piezoelectric discs are bonded to the aluminium alloy plate through the two-component epoxy paste adhesive, which isAW106. This AW106 is a room temperature curable adhesive, it can be cured at room temperature to obtain good bonding performance. The properties of the adhesive cured at 25 °C for 16 hours are shown in Table 4, the testing temperature is 25 °C.

**Figure 11:**
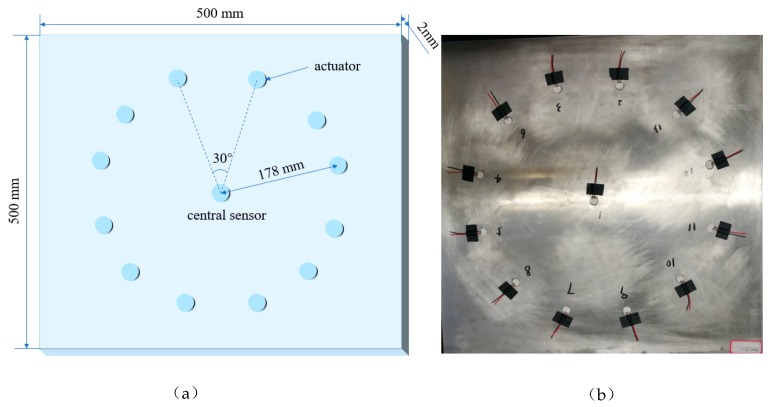
Layout of piezoelectric actuator/sensor on the aluminum plate: (**a**) layout design of piezoelectric elements; (**b**) actual placement of piezoelectric elements.

During service, both the excitation actuator and the receiving sensor may be debonded. Figure 11 shows the layout of the piezoelectric element debonding test. The influence of debonding area on monitoring signals was studied by changing the debonding area of peripheral actuators and center PZT sensors. A total of 13 PZT discs are bonded on the aluminum plate, 12 of them are placed around the aluminum plate as actuators, the rest one disc is bonded on the center of the aluminum plate as sensor. Among the 12 actuators bonded to the edge of the aluminum plate, the debonding areas of 10 actuators are set as 0%, 10%, 20%, 30%, 35%, 40%, 45%, 50%, 60%, and 70%, the other two are tested repeatedly as contrast samples. The ranges of debonding area of sensor bonded in the center of aluminium sheet are 0%, 20%, 40% and 60%. Considering the crosstalk between S0 mode and A0 mode, the distance between the actuators and sensors is set to 178 mm.

In order to obtain better adhesion properties between the piezoelectric element and aluminum plate, the experiment must be done in strict accordance with certain procedures, the bonding process of the piezoelectric sensor is as follows:

(1) The surface of the aluminum alloy sheet is pretreated prior to bonding, and the strength and durability of the bond are determined by proper bonding surface pretreatment. The surface of the aluminum plate is first cleaned with isopropyl alcohol to remove all oil, stains, and dust, and then sanded on the surface of the aluminum alloy to obtain the highest strength and durability of the bonded parts. After grinding, use isopropyl alcohol to perform a secondary cleaning process.

(2) Mix the A glue (resin) and B glue (curing agent) of epoxy resin AB at a ratio of 2:1 and stir for 50 s, the color of the adhesive gradually turns to milky white. The mixed adhesive should be used within 100 minutes.

(3) A thin layer of adhesive is covered on the non-lead side of the piezoelectric piece. The piezoelectric piece is bonded to the aluminuim alloy sheet by pressing the piezoelectric piece with uniform force on the fingertip for 90 s. It is best to observe that there is no adhesive overflow around the piezoelectric sheet. In this process, the debonding area and thickness of the adhesive are controlled by inserting PTFE film into piezoelectric disc and aluminum alloy plate.

(4) The aluminum alloy plate with piezoelectric pieces was placed at room temperature (25 °C) and cured for more than 24 h.

## 4. Results analysis

### 4.1. Effect of Debonding Area in Different Percentages on Signal

**Figure 12:**
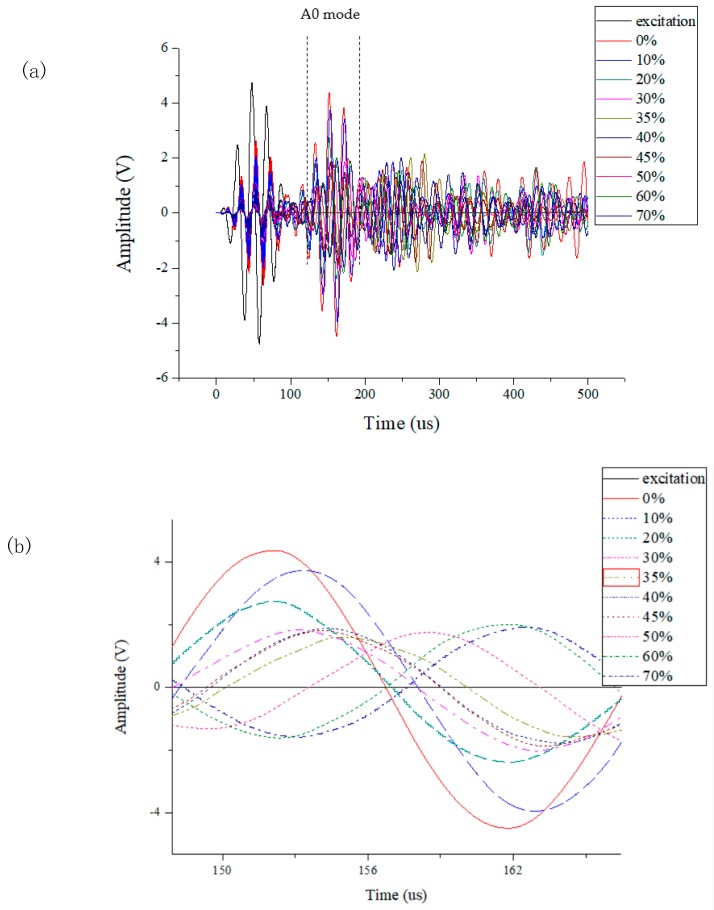
Signal data from the PZT elements at the condition that the central sensor is not debonded and the debonding area of peripheral actuators increases sequentially: (**a**) Lamb wave signal monitored by the system; (**b**) the time window of the A0 mode Lamb wave.

The piezoelectric element debonding monitoring system built in Section 2.2 is used to collect the Lamb wave signals monitored by the PZT under different debonding areas. Figure 12a is a schematic diagram of the Lamb wave signal monitored by the system at the condition that the central sensor without debonding, the debonding area of peripheral actuators increases gradually. Figure 12b shows the time window signal of the A0 mode taken from Figure 12a. As can be seen from Figure 12b, with the increasing of debonding area, the signal amplitude decreases and the signal curve shows a certain degree of shift.

**Figure 13:**
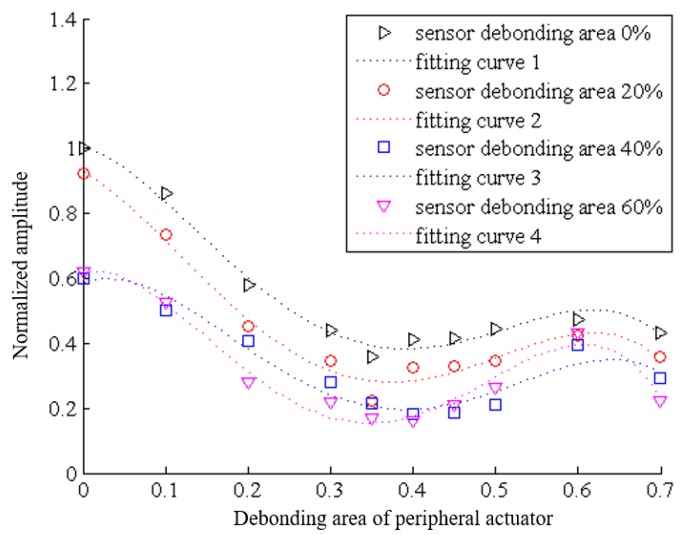
When the debonding area of the central sensor is 0%, 20%, 40% and 60%, the changes of normalized amplitude of the signal with the increase of the debonding area of the peripheral actuator, the excitation frequency is 50 kHz.

In order to observe the trend of Lamb wave more clearly under different debonding condition, the characteristic parameters, such as the normalized amplitude and phase shift of Lamb wave. In Figure 13 are the results of test-piece performed on central sensor having a debonding area of 0%, 20%, 40% and 60%,respectively, the peripheral actuators having debonding areas of 0%, 10%, 20%, 30%, 35%, 40%, 45%, 50%, 60% and 70%. The reference signal is the signal monitored when the excitation actuator and the receiving sensor are not debonded. For the Figure 13, fitting curve 1 represents the change of normalized amplitude when the central sensor debonding area is 0% and the debonding areas of actuator are from 0% to 70%. And so to fitting curve 2, 3 and 4, which represent debonding areas of sensor are 20%, 40% and 60% respectively. It is clear that line 1 to 4 show the same change trend, which indicated that whatever the debonding area of central sensor is, the increase of debonding area of peripheral actuator has the same effect on monitoring signal. Besides, from fitting curve 1 to fitting curve 3, the curves show a significant decline, while the trend from fitting curve 3 to curve 4 is smaller, which indicated that in the early stage of debonding of the central sensor, the normalized amplitude decreases by a large gradient and gradually decreases at the later stage. In addition, when the debonding area of the central sensor remains unchanged (0%, 20%, 40% and 60%), the normalized amplitude of the signal shows an overall downward trend with the increase of the debonding area of the peripheral actuators from 0% to 70%. The all curves of normalized amplitude of the signals show a quick drop in the early stage at the 35% turning point of actuator debonding, and then a slow rise to the 60% turning point before a slight drop. The first turning point of 35% may be due to the coincidence of the excited frequency of peripheral actuators with the inherent frequency of the central piezoelectric sensor, and the 60% \may be the result of the maximum ability of piezoelectric sensor to monitor signals.

**Figure 14:**
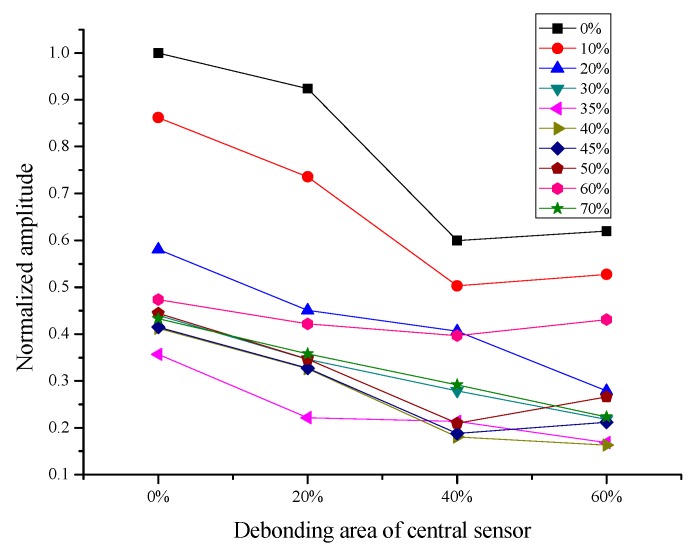
When the debonding area of the peripheral actuator is 0%, 10%, 20%, 30%, 35%, 40%, 45%, 50%, 60% and 70%, the changes of normalized amplitude of the signal with the increase of the debonding area of the central sensor, the excitation frequency is 50 kHz.

Figure 14 shows the change of normalized amplitude of the signal with the increasing of the debonding area of the central sensor when the peripheral actuator debonding area are 0%, 10%, 20%, 30%, 35%, 40%, 45%, 50%, 60% and 70%. As can be seen from the figure, the normalized amplitude shows a downward trend with the increase of central sensor debonding area. In the case that the peripheral actuators have small debonding areas such as 0% and 10%, the normalized amplitudes decrease rapidly. While the debonding area of the peripheral actuator is large, the normalized amplitude of the signal decreases slowly. Besides, when the debonding area of peripheral actuators achieves 35%, the value of normalized amplitude of the signal is the lowest overall. It is indicated that the debonding behavior of actuator has the greatest impact on structural health monitoring system when the debonding area is 35%.

**Figure 15:**
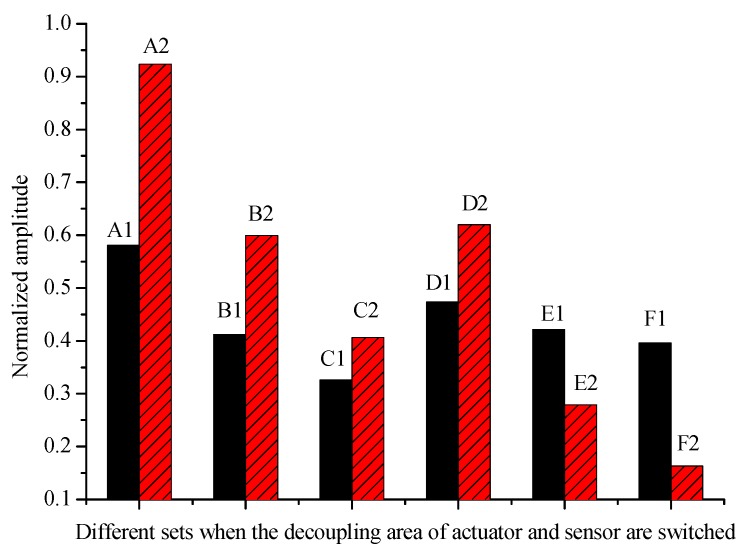
The normalized amplitude of the signal changes when the actuator and the sensor are decoupled.

In order to discuss the influence of the debonding of the excitation actuators and the receiving sensors on the monitoring signal, the condition the excitation actuators and the receiving sensors were respectively debonded was analyzed. Figure 15 shows the difference in monitoring signals when exchanging the debonding area of the actuator and the sensor. Six different combinations are set up, namely Ai to Fi (I = 1,2). The meaning of Ai to Fi is shown in Table 5. For example, 20%/0% of A1 represents 20% of the debonding area of the actuator and 0% of the sensor. While 0%/20% of A2 represents 0% of the debonding area of the actuator and 20% of the sensor. From the combination Ai-Ci, it can be seen that when the debonding area of the actuator and the sensor is less than 40%, the debonding of the actuator has a greater influence on the monitoring signal than the sensor. For the combination Ai, which contains the set 20%/0% and set 0%/20%, the amplitude of the monitoring signal when the sensor is debonded is much larger than that when the actuator is debonded. For the combination Bi and Ci, the amplitude of the signal when the sensor debonds is larger than that of the actuator which is similar to the combination Ai, but the degree is not as large, ss for the combination Di-Fi, the amplitude of the monitoring signal increases when the debonding area of the actuator exceeds 35% (as shown in Figure 13). For combination Di, the amplitude of D1 is larger than D2, that is to say, the influence of debonding of actuator on signal is greater than that of receiving sensor. For the combination Ei and Fi, in the case that the debonding area of the actuator and the receiving sensor are large, the debonding behavior of receiving sensor plays a dominant role in the influence of monitoring system. The above results indicate that in the structural health monitoring system, especially in the early stage of debonding of the PZTs, controlling the debonding of the actuator is very important for the reliability of the entire system.

**Figure 16:**
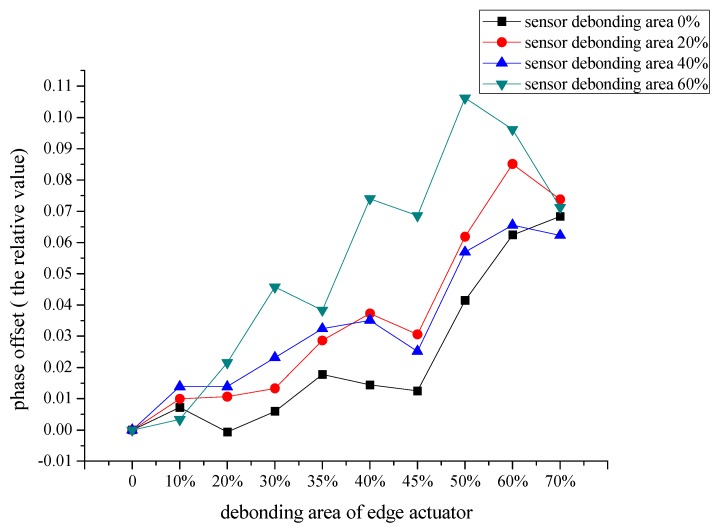
When the debonding area of the central sensor is 20%, 40% or 60%, the change of signal phase difference with the increase of the debonding area of the peripheral actuator, the excitation frequency is 50 kHz.

Figure 16 shows the change of the phase difference of monitoring signals with the increase of debonding areas of the peripheral actuators from 0% to 70% when the debonding areas of the central sensor are 0%, 20%, 40% and 60%. From the figure we can see that: (1) When the debonding area of the center sensor changes from 0% to 60%, the phase difference of the signals increases simultaneously, and the phase difference increase slowly at the beginning of central sensor debonding, while the curve corresponding to the debonding area of central sensor is 60% is far from the other three curves. The results indicate that the phase difference of monitoring signals showed a slow increase first and then rapidly with the increasing of debonding area of the central sensor. (2) When the debonding area of peripheral actuators change from 0% to 70%, the curves corresponding to the debonding areas 0%, 20%, 40% of central sensors show a slowly increase before the debonding area of peripheral actuator reaches around 45%, and then increase sharply with the increase of debonding of peripheral actuator. Nevertheless, the curve corresponding to the debonding area 60% of central sensor increases sharply all the time.

### 4.2. Effect of Excitation Frequency on Monitoring Signal

The influence of excitation frequency on the normalized amplitude of the signal was also investigated. As shown in Table 6, the excitation frequencies were set to 50 KHz, 60 kHz and 70 kHz, the central receiving sensor is completely bonded to the substrate, the debonding area of peripheral actuators ranges from 0% to 70%. The signal monitored when the central receiving sensor and the peripheral excitation actuator are bonded is taken as the reference signal. 

**Figure 17:**
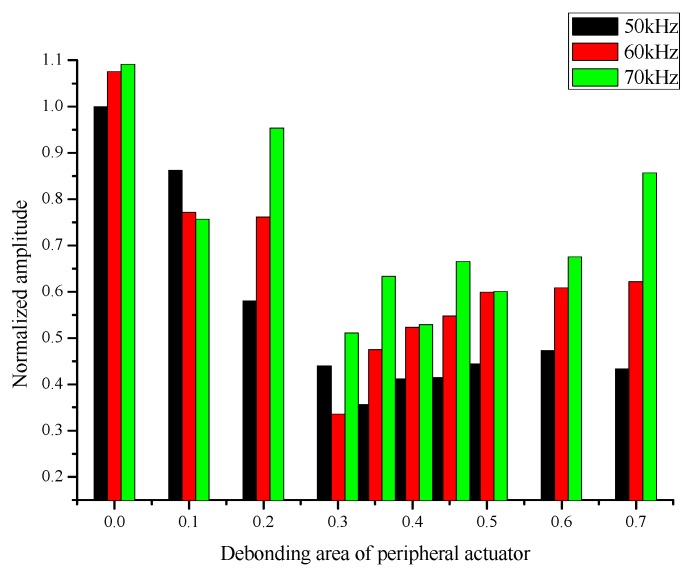
The variation of normalized amplitude of the signal when the excitation frequencies is 50 kHz, 60 kHz and 70 kHz.

In order to discuss the change trend of the normalized amplitudes of the signals at different excitation frequencies, the chart of the normalized amplitude of piezoelectric signals at the excitation frequencies of 50 kHz, 60 kHz and 70 kHz was depicted. As shown in Figure 17, the black bars, red bars and green bars correspond to the normalized amplitudes of the signals monitored at excitation frequencies of 50 kHz, 60 kHz and 70 kHz, respectively. From Figure 17 we can know that when the peripheral actuator and central sensor are full bonded on the aluminum plate, the increase of excitation from 50 kHz to 70 kHz lead to a rise of normalized amplitude. Whereas at the condition that the actuator and sensor are debonded simultaneously, the influence of excitation frequency is hard to draw a definite conclusion. The change tendency of normalized amplitudes shows the same at different excitation frequencies of 50 kHz, 60 kHz and 70 kHz. The test exhibited that the normalized amplitudes of monitoring signals reach the lowest point at the debonding area of actuator around 30%–35% at different frequencies. This may be the result that the excitation frequency of the peripheral actuator reaches the resonance frequency of the central receiving sensor.

## 5. Conclusions

A systematic experimental investigation of actuators and sensors debonding simultaneously in different percentages of debonding was designed to simulate the debonding damage of the piezoelectric elements in structural health monitoring (SHM) systems. An actuator/sensor layout was arranged in a central sensor with 12 peripheral actuators during the debonding tests. The signal-based damage monitoring method was used to analysis the monitored signals. The debonding of the actuators and the receiving sensors were designed in a wide range that a central sensor debonding areas were controlled to 0%, 20%, 40% and 60%, whereas the debonding areas of actuators were set from 10% to 70%. The excitation frequencies of the sensor were set to 50 kHz, 60 kHz and 70 kHz. The following results can be drawn from the analysis: 

(1) Both the debonding of excitation actuators and receiving sensors have a negative effect on the monitoring signals. On the condition that the debonding area of the center sensor changes from 0% to 60%, it is observed that the normalized amplitude decreases sharply first, and then slowly drop. When the debonding area of the central sensor is fixed (each with 0%, 20%, 40% and 60% respectively), the normalized amplitude of the signal shows an overall downward trend with the increase of the debonding area of the peripheral actuators from 0% to 70%. All depicted normalized amplitude curves show a quick drop in the early stage at the 35% turning point of actuator debonding, and then a slow rise to the 60% turning point before a slight drop. The first turning point of 35% may be due to the coincidence of the excited frequencies of peripheral actuators with the inherent frequency of the central piezoelectric sensor, and the 60% may be the result of the maximum ability of piezoelectric sensor to monitor signals.

(2) In the early stage of debonding, a significant decrease in the normalized amplitude of received signals will be exhibited for piezoelectric elements of both sensors and actuators. The degree of drop in normalized amplitude when actuators are debonded is much larger than that when the sensor is debonded. Therefore, in the structural health monitoring system, controlling the debonding behavior of piezoelectric element, especially the actuator’s debonding in the early stage is very important for the reliability of the entire SHM system. 

(3) The different degree of debonding of peripheral actuators and central sensor would lead to different phase angle offset of receiving signals. When the debonding area of the center sensor changes from 0% to 60%, the phase difference of the signals increases simultaneously, and such phase offset increases slowly at the beginning of central sensor debonding and will increases sharply when the central sensor debonds seriously up to 60% of the debonding area. Looking at the debonding areas of peripheral actuators ranging from 0% to 70%, the curves corresponding to the debonding areas 0%, 20%, 40% of central sensors show a slow increase, until the debonding area of peripheral actuator reaches around 45%, and increase sharply afterwards. Nevertheless, the curve corresponding to the debonding area 60% of central sensor exhibited the sharpest phase differences. 

(4) The change tendency of normalized amplitudes shows the same at different excitation frequencies of 50 kHz, 60 kHz and 70 kHz. The test exhibited that the normalized amplitudes of monitoring signals reach the lowest point at the debonding area of actuator around 30%–35% at different frequencies. This may be the result that the excitation frequency of the peripheral actuator reaches the resonance frequency of the central receiving sensor. 

## Figures and Tables

**Table 1:** The corresponding values of debonding areas and Ɵi (I = 1, 2, …, 9) when the debonding areas of actuator set from 10% to 70%.

Debonding Area (Percentage, %)	10	20	30	35	40	45	50	60	70
**Ɵ_i_ (°)**	93.6	120.6	142.2	153	162	171	180	198	217.8
**Debonding area (mm^2^)**	11.31	22.62	33.93	39.58	45.24	50.89	56.55	67.86	79.17

**Table 2:** 2024-T3 aluminum alloy performance parameters.

Material	Material	Tensile Strength (MPa)	Modulus of Elasticity (MPa)
2024-T3 aluminuium alloy	≥245	≥390	72,000

**Table 3:** Performance parameters of piezoelectric sensors.

**Product number**	SMD12T06R412WL
**Material**	SM412
**Dimensions**	12 mm x 0.6 mm
**Resonant frequency**	3.4 MHz ± 5%
**Resonant impedance**	≤6 Ω
**Static capacitance**	2.5 nF ± 30%
**Test Condition**	25 ± 3 °C; 40~70% R.H (Relative Humidity)

**Table 4:** Typical properties of adhesive cured at 40 °C for 16 hours (testing temperature 25 °C).

Model Numbers	Glass Transition Temperature (°C)	Bending Strength (MPa)	Flexural Modulus (MPa)	Floating Roll Peeling Test (N/mm)
AW106/HV9533U	45	60.4	1904.1	5

**Table 5:** Combination of actuator and sensor with different debonding area.

Group	Debonding Area of Actuator/Debonding Area of Sensor
A1	20%/0%
A2	0%/20%
B1	40%/0%
B2	0%/40%
C1	40%/20%
C2	20%/40%
D1	60%/0%
D2	0%/60%
E1	60%/20%
E2	20%/60%
F1	60%/40%
F2	40%/60%

**Table 6:** The variation of normalized amplitude of the signal when the excitation frequencies is 50 kHz, 60 kHz and 70 kHz.

	Freq. (kHz)	50	60	70
Debonding Area (%)				
**0**		1	1.0752	1.09156
**10**		0.86187	0.77144	0.75658
**20**		0.58094	0.76084	0.9537
**30**		0.43975	0.33556	0.5114
**35**		0.35679	0.47518	0.63314
**40**		0.41201	0.52282	0.52868
**45**		0.41481	0.54834	0.66513
**50**		0.44436	0.59944	0.60057
**60**		0.47364	0.6082	0.6754
**70**		0.43308	0.62191	0.85644
